# Successful treatment of a child with vascular pythiosis

**DOI:** 10.1186/1471-2334-11-33

**Published:** 2011-01-29

**Authors:** Tavitiya Sudjaritruk, Virat Sirisanthana

**Affiliations:** 1 Department of Pediatrics, Faculty of Medicine, Chiang Mai University, Chiang Mai, Thailand; 2 Research Institute for Health Sciences, Chiang Mai University, Chiang Mai, Thailand

## Abstract

**Background:**

Human pythiosis is an emerging and life-threatening infectious disease caused by *Pythium insidiosum*. It occurs primarily in tropical, subtropical and temperate areas of the world, including Thailand. The aim of this report is to present the first pediatric case of typical vascular pythiosis.

**Case Presentation:**

A 10-year-old boy with underlying β-thalassemia presented with gangrenous ulcers and claudication of the right leg which were unresponsive to antibiotic therapy for 6 weeks. Computerized tomography angiography indicated chronic arterial occlusion involving the right distal external iliac artery and its branches. High-above-knee amputation was urgently done to remove infected arteries and tissues, and to stop disease progression. Antibody to *P. insidiosum *was detected in a serum sample by the immunoblot and the immunochromatography tests. Fungal culture followed by nucleic sequence analysis was positive for *P. insidiosum *in the resected iliac arterial tissue. Immunotherapeutic vaccine and antifungal agents were administered. The patient remained well and was discharged after 2 months hospitalization without recurrence of the disease. At the time of this communication he has been symptom-free for 2 years.

**Conclusions:**

The child presented with the classical manifestations of vascular pythiosis as seen in adult cases. However, because pediatricians were unfamiliar with the disease, diagnosis and surgical treatment were delayed. Both early diagnosis and appropriate surgical and medical treatments are crucial for good prognosis.

## Background

Pythiosis is a life-threatening infectious disease occurring in humans and animals. The etiologic agent of the disease is *Pythium insidiosum*, an aquatic fungus-like organism (kingdom Chromista, phylum Oomycota, class Oomycetes), having an ecologic preference for swampy areas and found primarily in tropical, subtropical and temperate areas of the world [1-3].

Human pythiosis was first reported from Thailand in 1985 [4]. There are 4 different clinical forms of human pythiosis: vascular, ocular, cutaneous/subcutaneous and miscellaneous forms which account for 59, 33, 5, and 3% of all reported cases, respectively. All except the ocular form have been associated with patients who have underlying hematological diseases [2,3,5]. Human pythiosis is rare in children. Only a few well-documented cases have been reported, all of which were the cutaneous/subcutaneous form [6-8]. Human pythiosis has significant morbidity and mortality [2-5] because clinical information and diagnostic tools are limited, leading to under-recognition and under-diagnosis of the disease. Both early diagnosis and appropriate treatment are crucial for good prognosis. In this report, we described the first case of typical vascular pythiosis in a child in whom therapy was successful.

## Case presentation

A 10-year-old boy who had had a splenectomy for his underlying β-thalassemia was admitted to the Chiang Mai University (CMU) Hospital for a gangrenous ulcer on his right leg and claudication. Six weeks prior to admission, he first noticed a small pustular lesion on his right ankle which later spontaneously ruptured and became an ulcer. There was no previous history of trauma. The patient reported frequently working in a rice field with his parents. He was brought to a local hospital where the diagnosis of pyomyositis was made. Incision and drainage were performed and his symptoms improved. Two weeks prior to admission another pustular lesion developed on his right shin which finally became a gangrenous ulcer. His other symptoms were fever, severe resting pain, intermittent claudication and swollen right knee. He was transferred to a district hospital where an ultrasound showed thrombosis of the right femoral and popliteal artery and enlarged lymph nodes at the right popliteal fossa. Arthrocentesis of the right knee revealed 855,000 cells/mm^3 ^of WBC (polymorphonuclear cell 74%, mononuclear cell 26%). He was diagnosed as having septic arthritis. Intravenous cloxacillin was given and the patient was transferred to the CMU Hospital.

**Figure 1:**
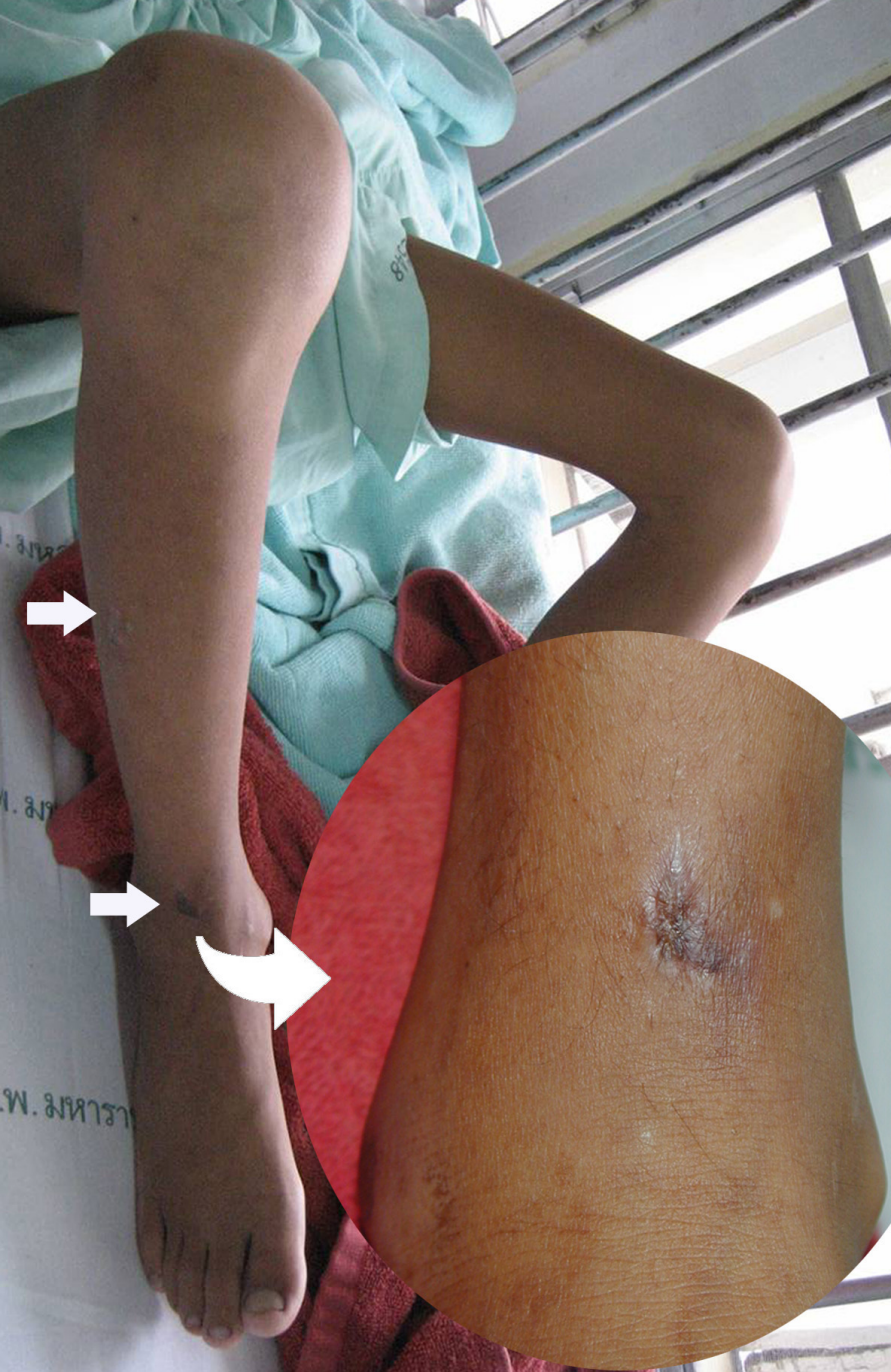
**Ulcerated scar on the right ankle and shin**.

**Figure 2:**
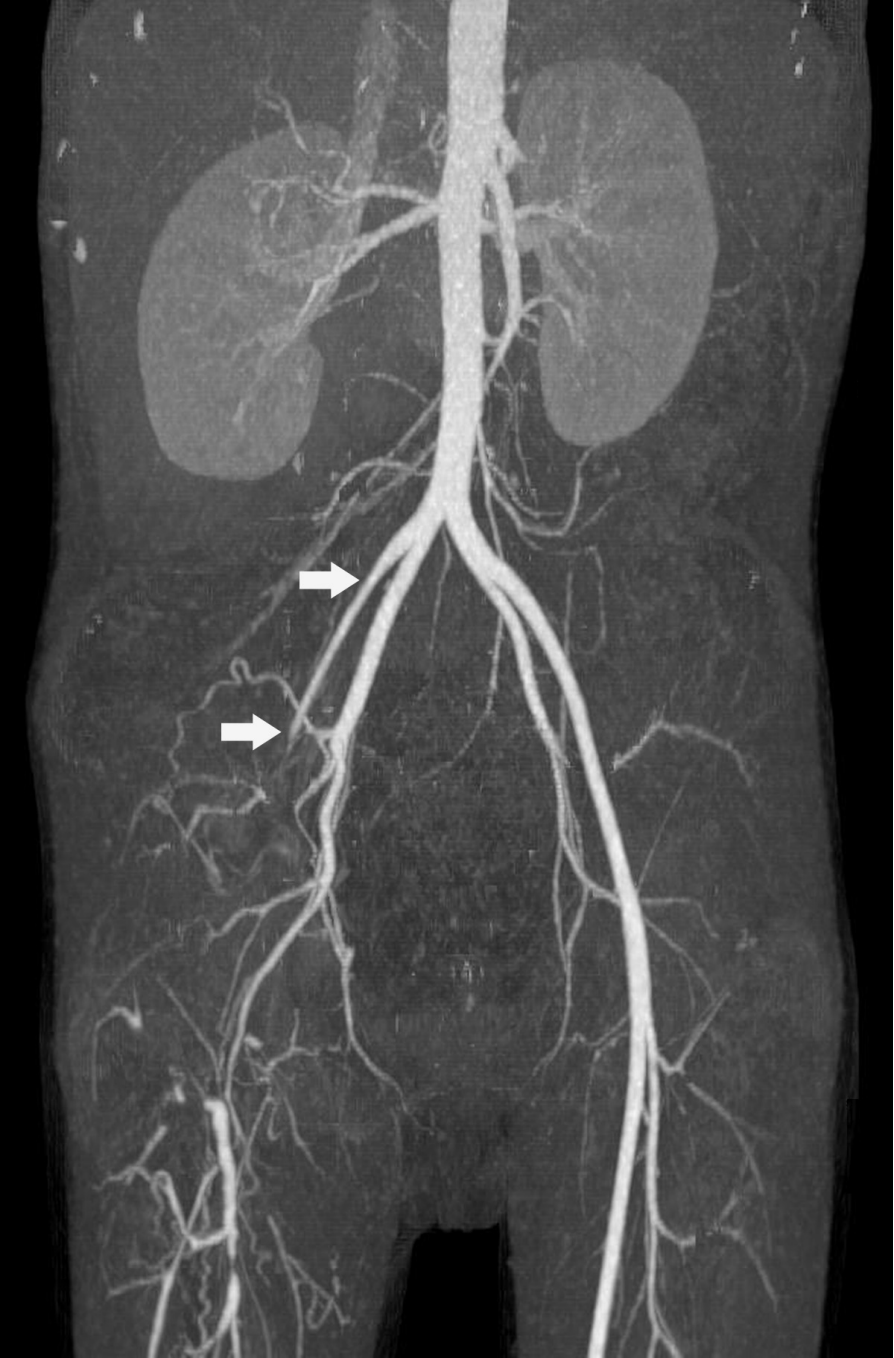
**Computerized tomography angiography of the lower extremities showed occlusion of the lower third of the right external iliac artery and its branches**.

Physical examination at the CMU Hospital revealed old ulcerated scars on the right shin and ankle (Figure 1), marked tenderness along the right leg, cool skin, diminished pulses of the right femoral and popliteal arteries, and absence of pulses of the right posterior tibialis and dorsalis pedis arteries. Computerized tomography angiography (CTA) showed occlusion of the lower third of the right external iliac artery, as well as the right common femoral, superficial femoral, popliteal, anterior tibial, tibioperonial, peroneal and dorsalis pedis arteries (Figure 2). Multiple collateral vessels were seen around the right knee. He was diagnosed as a case of vascular pythiosis and had a high-above-knee amputation of his right leg.

Antibody to *P. insidiosum *was detected in a serum sample by immunoblot and immunochromatography tests (ICT). Fungal culture followed by nucleic sequence analysis of 18S rRNA [9] was positive for *P. insidiosum *in the resected iliac arterial tissue. The tissue histopathology revealed evidence of chronic vasculitis with granulation tissue and thrombus on the anterior tibialis and the posterior tibialis up to the proximal margin of femoral arteries, but the special stains failed to demonstrate any etiologic agent, including *Pythium spp*.

After the surgery, the patient was given *P. insidiosum *immunotherapeutic vaccine (provided by Ariya Chindamporn, Chulalongkorn University, Bangkok; CUH Lot 140207/15-302), as well as the antifungal drugs, terbinafine at a dosage of 125 mg twice daily (13 mg/kg/day) and itraconazole at a dosage of 80 mg twice daily (8 mg/kg/day) for 2 months. The patient remained well and was discharged after almost 2 months after admission. Follow-up CTA performed 10 weeks after the amputation did not reveal recurrence of the disease. At the time of this communication, the patient has been symptom-free for 2 years.

## Conclusions

We report a case of thalassemic boy with vascular pythiosis who was successfully treated by surgery together with *P. insidiosum *immunotherapeutic vaccine and antifungal therapy.

Many cases of human pythiosis have been reported from Thailand [2-5]. Most cases were males between 20-60 years of age, working in agriculture [2,3]. Virtually all vascular pythiosis patients usually present with chronic arterial insufficiency syndrome of the lower extremities, which varies from intermittent claudication to gangrenous ulceration [2,3]. A history of exposure to a swampy area prior to the illness is common. Patients usually have underlying hematological diseases, most frequently thalassemia or paroxysmal nocturnal hemoglobinuria [2,3,5]. A typical vascular pythiosis has never been reported in a pediatric patient. Our child presented with the classical manifestations of vascular pythiosis as seen in adult cases. However, because pediatricians were unfamiliar with the disease, diagnosis and surgical treatment were delayed. If a timely diagnosis had been made, he could have been treated by a below-knee amputation which is associated with a much better post-operative function.

Culture identification of *P. insidiosum *is a definite diagnostic method for pythiosis [3]. *P. insidiosum *can be cultured and grows very fast in conventional nutrient agar for fungus. An induction of the zoospore formation is needed to confirm the identity of the organism [10]. However in cases of vascular pythiosis, the fungal culture is usually negative because viable fungus is only present at the advancing edge of the diseased artery which is difficult for the surgeon to obtain [1,2,4,10]. Among available serologic tests, all except the immunodiffusion test have high levels of sensitivity and specificity. The advantage of ICT and hemagglutination over other serological tests is that these tests are user-friendly and rapid [11-15]. PCR amplification and identification of the 18s rRNA gene of *P. insidiosum *are also useful for organism identification in both the clinical specimens and culture specimens [9,16].

Imaging studies, such as angiography and CTA, usually demonstrate an occlusion or aneurysm in medium-to-large-sized arteries of the lower extremities and trunk. They may also appear in collateral arteries due to the chronic course of the disease [2,3]. Histopathological studies may reveal fungal hyphae invading the arterial wall, with eosinophil infiltration, focal suppurative granuloma, as well as a blood clot containing hyphae in the arterial lumen [2]. Our patient presented with typical symptoms of vascular pythiosis. Antibody to *P. insidiosum *was detected by both the ICT and immunoblot methods. Fungal culture was positive for *P. insidiosum *in the resected iliac arterial tissue. His CTA study showed typical findings of vascular pythiosis. Although the histopathology of the surgical tissue of our case did not demonstrate any fungal hyphae, there was evidence of occlusion with a blood clot in the iliac artery.

Human pythiosis has a significant morbidity and mortality [2-5] because most healthcare professionals are not familiar with this rare organism and disease. Krajaejun *et al*. found that the overall mortality rate of human pythiosis was 29%, and increased to 40% in its vascular form [2]. Successful management of human pythiosis requires early recognition and appropriate treatment. In vascular pythiosis, surgical removal of the infected arteries, aneurysms, and surrounding tissues should be urgently carried out. Most cases undergo limb amputation [2,3] with careful attention paid to achieving an organism-free surgical margin. Because *Pythium spp*. does not possess ergosterol in its cytoplasmic membrane, conventional antifungal agents are ineffective for treatment of human pythiosis. However, there was one report of successful treatment of invasive facial pythiosis with two antifungal agents, namely terbinafine plus itraconazole [6]. Echinocandins, the new antifungal agents which inhibit the β-glucan synthesis of the cell wall of *P. insidiosum*, may be effective in the treatment of pythiosis [17]. An immunotherapeutic vaccine is another treatment option and favorable outcomes have been observed in humans [18].

In summary, human pythiosis is common in Thailand. It has high rates of morbidity and mortality. Early recognition requires the physician's awareness of the disease, especially in people with predisposing factors. Timely diagnosis and prompt treatment improve the prognosis.

## Consent

Written informed consent was obtained from the parents of the patient for publication of this case report and any accompanying images. A copy of the written consent is available for review by the Editor-in-Chief of this journal.

## Competing interests

The authors declare that they have no competing interests.

## Authors' contributions

TS and VS provided the clinical care of the reported patient and were involved in drafting the manuscript. Both provided the corresponding figures. All authors read and approved the final version of the manuscript.

## Pre-publication history

The pre-publication history for this paper can be accessed here:

http://www.biomedcentral.com/1471-2334/11/33/prepub

## References

[B1] Mendoza L Ajello L McGinnis MR Infection caused by the Oomycetous pathogen *Pythium insidiosum* J Mycol Med 1996 6 151 164

[B2] Krajaejun T Sathapatayavongs B Pracharktam R Nitiyanant P Leelachaikul P Wanachiwanawin W Clinical and epidemiological analyses of human pythiosis in Thailand Clin Infect Dis 2006 43 569 576 10.1086/506353 16886148

[B3] Krajaejun T Sathapatayavongs B Chaiprasert A Srimuang S Do you know human pythiosis J Infect Dis Antimicrob Agents 2008 25 45 51

[B4] Thianprasit M Chaiprasert A Imwidthaya P Human pythiosis Curr Top Med Mycol 1996 7 43 54 9504058

[B5] Sathapatayavongs B Leelachaikul P Prachaktam R Atichartakarn V Sriphojanart S Trairatvorakul P Human pythiosis associated with thalassemia hemoglobinopathy syndrome J Infect Dis 1989 159 274 280 2644370 10.1093/infdis/159.2.274

[B6] Shenep JL English BK Kaufman L Pearson TA Thompson JW Kaufman RA Successful medical therapy for deeply invasive facial infection due to *Pythium insidiosum *in a child Clin Infect Dis 1998 27 1388 1393 10.1086/515042 9868648

[B7] Triscott JA Weedon D Cabana E Human subcutaneous pythiosis J Cutan Pathol 1993 20 267 271 10.1111/j.1600-0560.1993.tb00654.x 8366216

[B8] Rinaldi MG Seidenfeld SM Fotherbill AM McGough DA *Pythium insidiosum *causes severe disease in healthy boy Mycol Observer 1989 9 7

[B9] Vanittanakom N Supabandhu J Khamwan C Praparattanapan J Thirach S Prasertwitayakij N Identification of emerging human-pathogenic *Pythium insidiosum *by serological and molecular assay-based methods J Clin Microbiol 2004 42 3970 3974 10.1128/JCM.42.9.3970-3974.2004 15364977 PMC516349

[B10] Chaiprasert A Samerpitak K Wanachiwanawin W Thasnakorn P Induction of zoospore formation in Thai isolates of *Pythium insidiosum* Mycoses 1990 33 317 323 2259373 10.1111/myc.1990.33.6.317

[B11] Pracharktam R Changtrakool P Sathapatayavongs B Jayanetra P Ajello L Immunodiffusion test for diagnosis and monitoring of human *pythiosis insidiosi* J Clin Microbiol 1991 29 2661 2662 1774283 10.1128/jcm.29.11.2661-2662.1991 PMC270400

[B12] Krajaejun T Kunakorn M Niemhom S Chongtrakool P Pracharktam R Development and evaluation of an in-house enzyme-linked immunosorbent assay for early diagnosis and monitoring of human pythiosis Clin Diagn Lab Immunol 2002 9 378 382 11874882 10.1128/CDLI.9.2.378-382.2002 PMC119942

[B13] Krajaejun T Kunakorn M Pracharktam R Chongtrakool P Sathapatayavongs B Chaiprasert A Identification of a novel 74-kiloDalton immunodominant antigen of *Pythium insidiosum *recognized by sera from human patients with pythiosis J Clin Microbiol 2006 44 1674 1680 10.1128/JCM.44.5.1674-1680.2006 16672392 PMC1479217

[B14] Jindayok T Piromsontikorn S Srimuang S Khupulsup K Krajaejun T Hemagglutination test for rapid serodiagnosis of human pythiosis Clin Vaccine Immunol 2009 16 1047 1051 10.1128/CVI.00113-09 19494087 PMC2708401

[B15] Krajaejun T Imkhieo S Intaramat A Ratanabanangkoon K Development of an immunochromatographic test for rapid serodiagnosis of human pythiosis Clin Vaccine Immunol 2009 16 506 509 10.1128/CVI.00276-08 19225072 PMC2668273

[B16] Grooters AM Gee MK Development of a nested polymerase chain reaction assay for the detection and identification of *Pythium insidiosum* J Vet Intern Med 2002 16 147 152 11899029 10.1892/0891-6640(2002)016<0147:doanpc>2.3.co;2

[B17] Cavalheiro AS Maboni G de Azevedo MI Argenta JS Pereira DI Spader T In Vitro activity of terbinafine combined with caspofungin and azoles against *Pythium insidiosum* Antimicrob Agents Chemother 2009 53 2136 2138 10.1128/AAC.01506-08 19289531 PMC2681559

[B18] Wanachiwanawin W Mendoza L Visuthisakchai S Mutsikapan P Sathapatayavongs B Chaiprasert A Efficacy of immunotherapy using antigens of *Pythium insidiosum *in the treatment of vascular pythiosis in humans Vaccine 2004 22 3613 3621 10.1016/j.vaccine.2004.03.031 15315840

